# Color stability of two different resin matrix ceramics: randomized clinical trial

**DOI:** 10.1186/s12903-023-03364-6

**Published:** 2023-09-14

**Authors:** Aliaa Ibrahim Mahrous, Aya A. Salama, Alshaimaa Ahmed Shabaan, Ahmed Abdou, Mohamed Mostafa Radwan

**Affiliations:** 1 Fixed Prosthodontic Department, Faculty of Dentistry, Fayoum University, Fayoum, Egypt; 2 Fixed Prosthodontics Department, Faculty of Dentistry, October University for Modern Sciences and Art, 6th of October City, Egypt; 3 Faculty of Dentistry, Fayoum University, Fayoum, Egypt; 4 Prosthetic Dentistry Department, Biomaterials Division, Faculty of Dentistry, King Salman International University, El Tur, South Sinai Egypt; 5 Faculty of Dentistry, Al-Ayen University, Thi-Qar, Iraq; 6 Fixed Prosthodontic Department, Faculty of Dentistry, Bani Suif University, Bani Suif, Egypt

**Keywords:** Dental restoration, Ceramics, Resin matrix, Color stability, Spectrophotometer

## Abstract

**Background:**

One of the most common causes of aesthetic failure and restoration replacement is the tooth restorations color mismatch specifically after aging.

**Methods:**

One hundred and two participants with endodontically treated first molar were selected clinically. The patients were randomly splited into two groups and restored either with Cerasmart hybrid ceramic or Vita Enamic polymer infiltrated ceramic network (PICN) crowns and cemented using dual cure adhesive cement. The color difference (ΔE) values after cementation at 0 (Baseline), 6, and 12 months of use were obtained by quantification of L*, a*, and b* values with a digital spectrophotometer. Mann–Whitney test used to compare between tested groups at each time point and between (α = 0.05).

**Results:**

At 6 months follow-up intervals, Vita Enamic group showed the highest significant ∆L* (*p* = 0.035) and ∆a* (*p* < 0.001) compared to Cerasmart group. ∆b* and ∆E showed no significant difference between both groups (*p* > 0.05). Furthermore, all color parameters of both groups showed statistically significant difference at 12 months follow-up intervals. After 12 months, Vita Enamic restorations presented higher color change compared to Cerasmart restorations with a (*p*-value of 0.0120). When comparing the total color difference ∆Et through-out the follow-up intervals of Vita Enamic & Cerasmart groups, there were insignificant difference (*p* = 0.263).

**Conclusion:**

Both hybrid materials demonstrated comparable color stability after 1 year of clinical service within clinical acceptance range. However, Cerasmart demonstrated a better colour stability after 1 year.

**Trial registration:**

ClinicalTrials.gov (ID: NCT05501808) 15/8/ 2022- ‘retrospectively registered’.

## Background

The aesthetic CAD/CAM materials have undergone significant advancements, changing the treatment from a two-step, bi-layered, high-strength ceramic restoration to a single step, monolithic restoration that eliminates veneering chipping and fracture issues [1, 2]. Obtaining the ideal optical qualities of natural teeth with artificial restorative materials is one of modern dentistry’s primary challenges. Nowadays, fixed dental prosthesis can be made from a wide range of dental materials. Ceramics and modified composites are largely in question for this purpose, as they are manufactured to closely resemble natural teeth in color, which is a desirable trait for many patients [3–6]. Ceramics have been the preferred material due to their exceptional aesthetics, high strength, and biocompatibility. However, ceramic restorations are challenging as it can cause opposing teeth to wear out excessively and have poor repair options [6].

With the advancements in adhesive dentistry and resin composites, a new unique material that has been referred to as “nano-ceramics” or “hybrid ceramics” in the commercial market was developed in the last ten years with high chance of success as aesthetic restoration [7]. A shock absorber hybrid ceramic is a modern alternative to traditional ceramics for use in dental restorations such as inlays, partial crowns, and full crowns [8, 9] with physical qualities highly comparable to those of teeth [10]. Hybrid ceramics have properties between those of ceramics and composites [11, 12], including somewhat high fracture toughness, elastic modulus, hardness, and rigidity. Hybrid ceramics are more flexible, less expensive, and less prone to fracture. Also, when compared to all ceramic materials and composites, it gives rise to reduced abrasion of opposing natural teeth with an important advantage over ceramics [13–17]. In addition to easier fabrication, being repaired easily and used right in the dentist’s chair without needing to be fired first [11, 18].

Hybrids are considered as infiltrating porous ceramic with polymers. There are two categories on the market based on microstructure; dispersed filler (DF) and polymer-infiltrated ceramic network (PICN). The ceramic and polymer phases of PICN, also known as resin interpenetrating network ceramic, form a continuous three-dimensional interconnected network [8]. A PICN example is Vita Enamic (VITA Zahnfabrik) while GC America’s Cerasmart is an example for resin with dispersed fillers [6, 9, 19].

The first material in this classification to be introduced is Vita Enamic. The product’s attributes, according to the manufacturer, lessen the likelihood of crack propagation and fracture by combining the advantages of composites and ceramics to relay strength and elasticity and behave like dentin. Its flexural strength ranges from 150 to 160 MPa and its composition is 86% ceramic and 14% polymer. Due to the special ceramic composition, it is unique in that it can be etched with 5% hydrofluoric acid for 60 s on the intaglio surface [8, 9].

Cerasmart nanoceramic block was introduced to combine the advantageous qualities of ceramic and resin technology. The material is made up of 29% resin and 71% silica and barium glass nanoparticles. This material, which has a flexural strength of 238 MPa, is intended for anterior, posterior, and implant restorations that require the least amount of preparation [6, 10].

Aesthetic considerations in dental rehabilitation highlight the importance of the color stability of dental materials. Different foods and beverages can stain dental restorations when they come into contact. All these may cause the restorations to fail aesthetically. Composites have a far higher discoloration potential when compared to ceramics [20, 21].

The amount of discoloration is mostly determined by the composition of the material, type of environment, and contact period [22]. The lightness, Hue, and chroma components of the traditional CIELab color formula are denoted by the letters L*, H*, and C respectively. Accordingly, L*, a* and b* where L* stands for lightness and takes a number [11] between 0 to 100, with 0 refers to black while 100 stands for white. The saturation on the letter a* is the saturation on the red-to-green axis, and b* blue-yellow axis. In order to calculate color differences, a formula is used to express these parameters as “E values” [23, 24].

In clinical situations with different dental materials, the evaluation of color distinctions is quite important. The color stability of resin matrix ceramics is critical, as clinically observable over time. Due to the high demand for cosmetic procedures, clinicians must be careful when selecting restorative materials, since this is one of the most important elements determining long-term treatment effectiveness [25].

The durability of color in contemporary hybrid ceramics is mostly unknown. The current in-vivo study aimed to probe the color stability of two hybrid ceramics (Vita Enamic; EN) and (Cerasmart; CS) under the variety of clinical conditions. The null hypothesis of the present study was that there will be no statistically significant difference regarding color stability of the tested two resin matrix ceramics.

## Materials & methods

The study was carried out at the Fixed Prosthodontics Department, Faculty of Dentistry, Fayoum University, between June 2021 and August 2022. The Declaration of Helsinki, which governs research involving human subjects, was followed [26]. The Fayoum University’s supreme ethics committee EC2140 gave the study protocol a thorough review, approval, and retrospective registration in the ClinicalTrials.gov database. (Identifier: NCT05501808) on 15th of August 2022. Prior to the study’s start, each participant provided their written, informed consent to share their data, and they were free to leave the study at any time without having to give a reason.

### Sample size

Based on previous research [27], a sample size calculation was made using STATA16 with a desired alpha of 0.05 and power of 80% to distinguish between the null hypothesis, which states that the means of both groups are 1.722, and the alternative hypothesis, which states that the means of the Vita Enamic hybrid ceramic group are 2.2, with estimated group standard deviations of 0.854 and the ratio of the sample sizes in groups to be 1. It was determined that 90 participants would make up the entire sample for this study. However, 102 participants (51 in each group), presuming potential dropouts, were included in this study.

### Study design and randomization

**Fig. 1:**
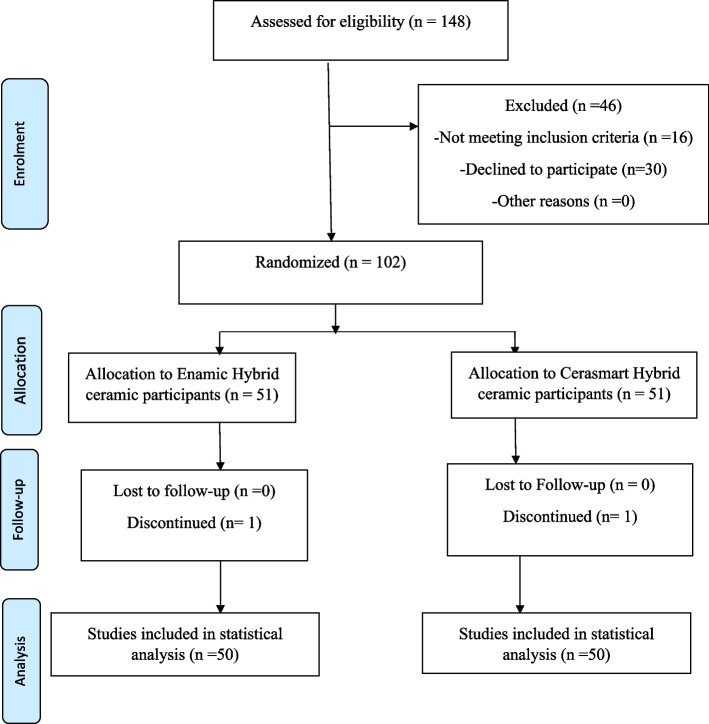
Consort flow chart

To ensure balance in the number of participants assigned to each group, this study was prepared as a double-blinded prospective randomized controlled trial. The randomization was unstratified and carried out via a random block design with blocks of size 2, 4, and 6 to ensure balance in the number of patients assigned to each group. The study was designed in accordance with the Consolidated Standards of Reporting Trials (CONSORT) statement of 2012 (Fig. 1) [28].

One hundred two participants with previous endodontically treated first molar were eligible for full coverage hybrid ceramic crown in lower 1^st^ molar. The inclusion criteria were as follows: (1) Age of the participants from 18–60 years old who can read and sign informed consent document. (2) Participants with proper endodontic treatment (3) participants with no systemic conditions that could affect the procedures. (4) No active periodontal disease. (5) Participants with stable normal occlusion. While the exclusion Criteria were; (1) Participants with high caries index. (2) Participants with smoking habits. (3) Participants with parafunctional habits as clenching/bruxism. (4) Participants with drugs that affect oral health. (5) Pregnant females.

The participants were allocated to one of the two groups based on hybrid ceramic type used, (Table 1). Vita Enamic group (*n* = 51): crowns were milled from (Vita Enamic, Vita Zahnfabrik, Bad Säckingen, Germany) while Cerasmart group (*n* = 51): crowns were milled from (Cerasmart; CS,GC Corp., Tokyo, Japan). The material used served as the primary predictor. The assessors and participants were both blinded for the duration of the study.

**Table 1:** Description of hybrid ceramics utilized in the study

**Hybrid system**	**Composition**	**Manufacturer**
GC Cerasmart (Resin matrix ceramic)	Resin matrix: Bis-MEPP, UDMA, dimethacrylate	GC Corp., Tokyo, Japan
	Inorganic filler: silica, barium glass (71.0 wt%)	
Vita Enamic (Resin matrix ceramic (polymerinfiltrated-ceramic-network (PICN))	Resin matrix: UDMA, TEGDMA	Vita-Zahnfabrik, Bad Sackingen, Germany
	Inorganic filler: feldspar ceramic enriched with aluminum oxide (86.0 wt%)	

*TEGDEMA* Triethylene glycol dimethacrylate, *Bis-MEPP* (2,2-Bis (4-methacryloxypolyethoxyphenyl) propane, *UDMA* urethane dimethacrylate)

### Intervention

All participant underwent clinical, radiographic evaluation of all cases with shade selection using Vita System 3-D Master (VITA) was performed. During the preparatory visit, any carious lesions were removed and restored using a core build-up restorative material (GC Fuji lining LC; GC Corp, Tokyo, Japan) upon requirements. All teeth were prepared in a standardized reduction parameters following the manufacturer’s recommendations with a deep chamfer finish line, 1–1.5 mm occlusal reduction and 0.8–1.5 mm axial reduction. Astringent retraction paste (VOCO GmbH) was used into sulcus prior to digital impression.

CEREC, Dentsply Sirona was used to scan and construct the entire crowns in current study. An intra-oral impression was captured using a powder-free intraoral scanner CEREC Omnicam (Dentsply Sirona) following the manufacturer’s recommendations. The prepared tooth, opposing teeth and interocclusal relationship was recorded and the biogeneric individual design mode of CAD software was used for the design. CEREC MCXL milling machine (Dentsply Sirona) was used for CAM fabrication of both groups. Checking of all crowns for proper fit with adjustments to occlusal and proximal contacts were made followed by final finishing with NTI CeraGlaze (Axis, Coppell) on an electric handpiece while following the manufacturer’s instructions at 10,000 rpm for 10 s with a fine finishing wheel and at 5,000 rpm for 10 s with a high-shine polishing wheel.

At delivery visit, all prepared teeth followed same protocol for cementation. All prepared teeth were cleaned using Pumice and Robinson brush before isolated with rubber dam. Phosphoric acid etchant 37% was applied for 20 s, washed and air dried & bonding agent was applied. The fitting surfaces of all crowns were etched 60 s using 5% hydrofluoric acid according to the manufacturer’s instructions. The etched surfaces were thoroughly water sprayed and oil-free dried before application of silane coupling agent for 60 s. Dual-cure resin cement (Vriolink Esthetic, Ivoclar Viadent AG, Schaan, Liechtenstein) was used. Using a sharp explorer, extra cement was scraped off. Air-blocking gel (Oxiguard II: Kuraray Noritake Dental Inc.: Tokyo, Japan) was finally applied and light-cured (Elipar Deepcure-S: 3 M Espe: St. Paul, USA) about 20 s.

A spectrophotometer (VITA Easy-shade Advance 4.0, VITA Zahnfabrik, Bad Säckingen, Germany) was used to assess the color of all participants at the follow-up appointments (baseline = 24 h after cementation, 6 months and 12 months). It was calibrated before each measurement. CIELAB parameters (L*, a*, b*) were recorded in at each follow-up appointment with the same operator who was blinded. L* indicated the material lightness, a* denotes hues change across the red-green axis, while b* showed the hues difference across the yellow-blue axis. Finally, ΔL*, Δa*, Δb*and ΔE were calculated for 6, 12 month and finally the overall color changes over the year. CIELAB (ΔE_ab_) was used for calculating the color differences as follows;

$$\begin{array}{l}{\Delta\mathrm{E}}_6=\lbrack{({\Delta\mathrm{L}}_6=\text{L}_6^\ast-\mathrm L_0^\ast)}^2+{({\Delta\mathrm{a}}_6=\mathrm a_6^\ast-\mathrm a_0^\ast)}^2+{({\Delta\mathrm{b}}_6=\mathrm b_6^\ast-\mathrm b_0^\ast)}^2\rbrack^{1/2}\\{\Delta\mathrm{E}}_{12}=\lbrack{({\Delta\mathrm{L}}_{12}=\mathrm L_{12}^\ast-\mathrm L_6^\ast)}^2+{({\Delta\mathrm{a}}_{12}=\mathrm a_{12}^\ast-\mathrm a_6^\ast)}^2+{({\Delta\mathrm{b}}_{12}=\mathrm b_{12}^\ast-\mathrm b_6^\ast)}^2\rbrack^{1/2}\\{\Delta\mathrm{E}}_{\mathrm t}=\lbrack{({\Delta\mathrm{L}}_{\mathrm t}=\mathrm L_{12}^\ast-\mathrm L_0^\ast)}^2+{({\Delta\mathrm{a}}_{\mathrm t}=\mathrm a_{12}^\ast-\mathrm a_0^\ast)}^2+{({\Delta\mathrm{b}}_{\mathrm t}=\mathrm b_{12}^\ast-\mathrm b_0^\ast)}^2\rbrack^{1/2}\end{array}$$

### Statistical analysis

Data checked for normality using Kolmogorov Smirnova test. Data displayed non-normal distribution, so Mann–Whitney test used to compare between tested group at each time point and between time points for each group. Significant level was set at *p* = 0.05. Statistical analysis was done using IBM SPSS (version 26, Armonk, NY, USA).

## Results

This study included 102 participants allocated into 2 groups equally. No complications were observed and no drop out except for one participant in each group which was excluded from the analysis. At 6 months follow-up intervals, Vita Enamic group showed the highest significant ∆L*, (*p* = 0.035) and ∆a*, (*p* < 0.001) compared to Cerasmart group. While, ∆b*, and ∆E showed insignificant difference between both groups (*p* > 0.05). Furthermore, all color parameters of Cerasmart showed lower significant values compared to Vita Enamic at 12 months follow-up intervals.

**Fig. 2:**
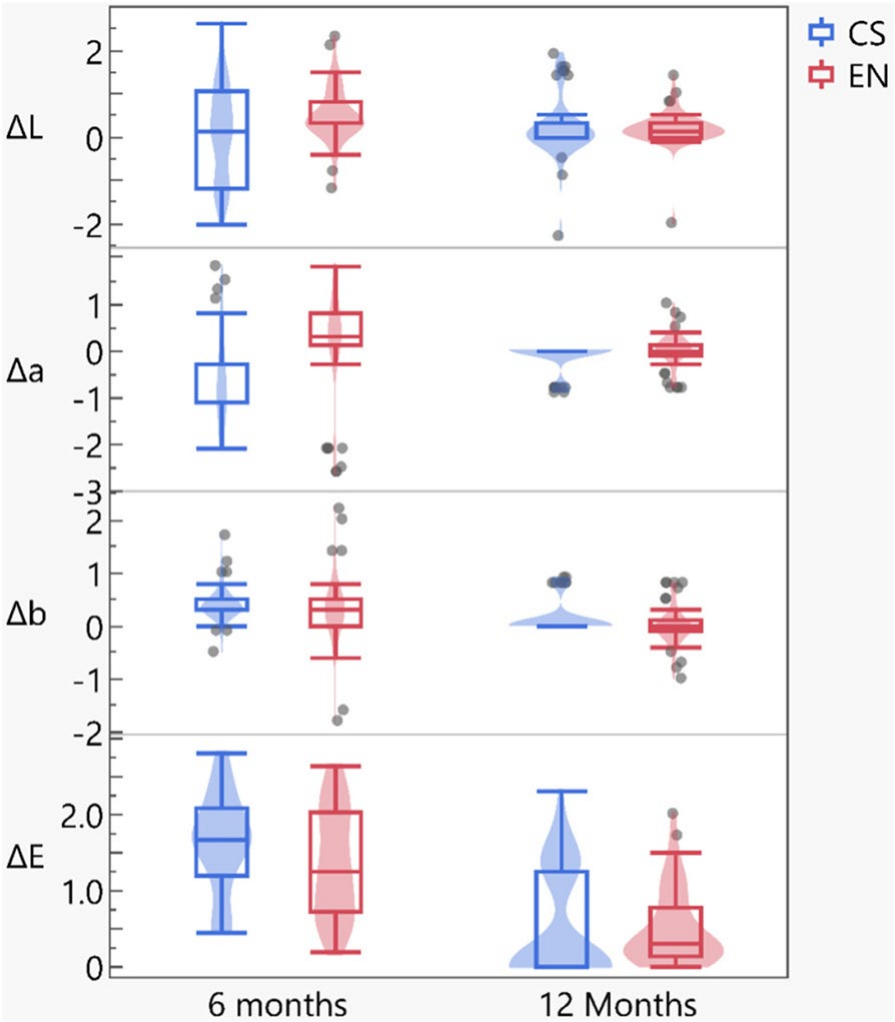
Box Plot showing comparison between Vita Enamic and Cerasmart in all Color parameters (∆L*, ∆a*, ∆b*&∆E) at follow-up intervals of 6month and 12 month

Regardless of the restoration materials, all color parameters showed significant decrease except for ∆L*, within Cerasmart group (Table 2 and Fig. 2).

**Table 2:** Mean [95% CI] for color parameters (∆L, ∆a, ∆b, and ∆E) at follow-up intervals for different tested groups

*Color parameter*	*Material*	*6 months*	*12 Months*	*p-value*
*∆L*	*EN*	*0.3[0.3 to 0.8]*	*0.1[0.0 to 0.3]*	< *0.001*
	*CS*	*0.1[-1.2 to 1.1]*	*0.0[0.0 to 0.3]*	*0.8488*
*p-value*		*0.0351*	*0.0216*	
*∆a*	*EN*	*0.3[0.1 to 0.8]*	*0.0[-0.1 to 0.1]*	< *0.001*
	*CS*	*-1.1[-1.1 to -0.3]*	*0.0[0.0 to 0.0]*	< *0.001*
*p-value*		< *0.001*	*0.0044*	
*∆b*	*EN*	*0.3[0 to 0.5]*	*0.0[-0.1 to 0.1]*	< *0.001*
	*CS*	*0.3[0.3 to 0.5]*	*0.0[0.0 to 0.0]*	< *0.001*
*p-value*		*0.1896*	*0.0044*	
*∆E*	*EN*	*1.2[0.7 to 2]*	*0.3[0.1 to 0.8]*	< *0.001*
	*CS*	*1.7[1.2 to 2.1]*	*0.0[0.0 to 1.2]*	< *0.001*
*p-value*		*0.0853*	*0.0120*	

Significant level was set at *p* < 0.05

**Fig. 3:**
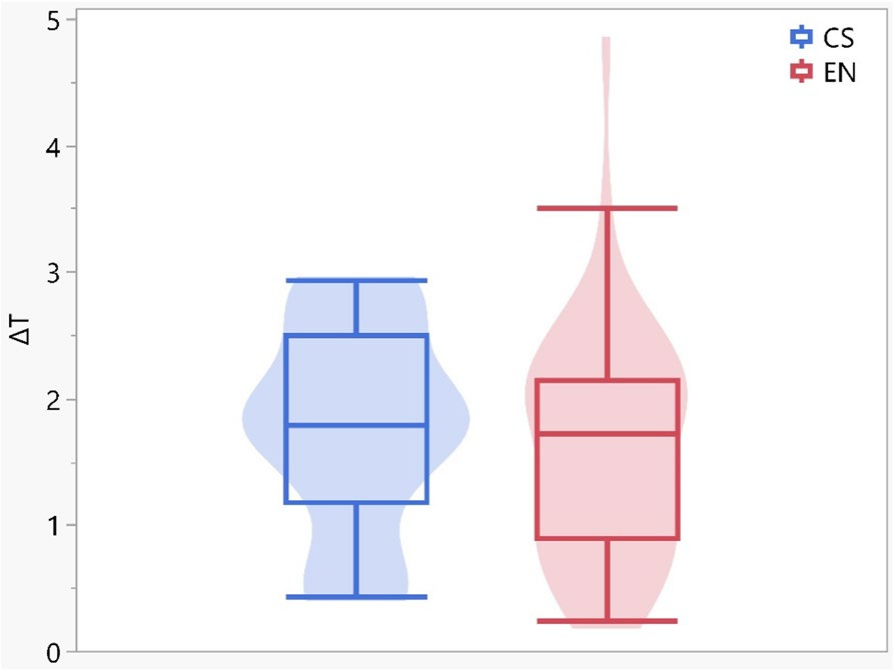
Box Plot showing the ∆E_t_ (total color difference after 1 year)

When comparing the total color difference ∆E_t_ through-out the follow-up intervals of Vita Enamic & Cerasmart groups, there were insignificant difference (*p* = 0.263) (Fig. 3).

## Discussion

Over the past few years, there has been a growing trend of patients requesting or expecting high aesthetic restoration. Color stability is an essential clinical aspect when it comes to the aesthetics which in turns affect the long-term clinical success of resin-based ceramic restorations. Plaque accumulation, solution stains, surface roughness, and chemical reactions can all affect the color of these materials [29, 30].

As instrumental color measurement has the advantages of being objective and quantified, CIE lab formula was selected in the current study to determine any color changes [31]. The color change values were represented by ΔE which is defined as the numerical distance between the L*a*b* coordinates of two colors. It was reported that there were a range before detectable color changes. ΔE < 1 were undetectable by the human eye; 1.0 < ΔE < 3.3 was seen only by a skilled person and considered clinically acceptable while, ΔE > 3.3 was considered easily observed and not acceptable clinically [31, 32].

Previous research on hybrid materials has mostly concentrated on mechanical qualities [33], making the characterization of optical properties rather limited. Based on the assumption that resin-based restorative materials are predicted to be less color-stable upon aging due to polymer matrix decomposition, unreacted monomers of the polymerization agents, and extrinsic staining agents, materials with comparable composition and fundamental microstructures were chosen for the current study [5, 34, 35]. Cerasmart and Vita Enamic are both categorised as hybrid materials with different compositions which justifies the necessity to study variations in color stability. Cerasmart material is made up of 29% cross-linked polymer blends and 71% ceramic-like inorganic silicate glass fillers. Contrarily, Vita Enamic is containing 86% feldspathic ceramic and 14% cross-linked polymer blends [36].

Currently, there is no published clinical trial research on the color stability of resin-ceramic infiltrated materials. Therefore, this study compared the color stability of two resin hybrid ceramic restorations in randomized clinical trial. Founded on the study outcomes, the null hypothesis stated was accepted since the color stability of Vita Enamic was comparable to that of Cerasmart hybrid ceramic; with no statistically significant difference after one year of clinical service and all was within the clinically acceptable range of color difference (ΔE > 3.3) [21, 32]. In contrast, Barutçug et al. [37] found that a discoloration greater than the clinically acceptable threshold level (∆E00 = 2.25) was seen for both Cerasmart and Vita Enamic after a month of exposure to beverages.

The results of the current study compared color alterations in two dental materials over a period of 6, 12 months and the total change. The findings showed that the Vita Enamic group significantly higher than the Cerasmart group in terms of ΔL* (lightness) and, Δa* (hue) values at 6-month intervals, whereas there was no significant difference in Δb* (color tone) and E (overall color difference) values between both groups with a *p*-value of 0.0853. This suggested that Vita Enamic in the short term have a more pronounced impact on the teeth’s brightness (lightness) and color (hue) than Cerasmart.

Difference in materials color stability across the research timeline could be endorsed to differences in composition and microstructure [36]. The higher color change values of Vita Enamic could be credited to its alumina content (8.31 wt%), that could cause decrease in translucency with less color stability compared to Cerasmart on the long term of use [38]. Contrary, Cerasmart has a lower proportion of alumina and increased ratio of zirconia and silica nanoparticles surrounded in a highly cross-linked resin matrix, which may explain its greater translucency and color stability [39]. Furthermore, the ageing process disrupts the chemical link between the filler and the resin matrix, which contributes to color instability [40].

Additionally, the sustainability of a restoration’s color is mainly influenced by the type of resin matrix, as water absorption by the resin component of the material is a major contributing factor to color changes [7, 41]. Hydrophilic bisphenol A-glycidyl methacrylate (BISGMA) is a methacrylate monomer with high strength and durability that is used commonly [42, 43]. It can cause discoloration due to the high susceptibility to leaching from the material. Paolone et al. [44] suggested also that this monomer is responsible for water uptake and, therefore, possibly for discoloration. Hydrophobic urethane dimethacrylate (UDMA) is another methacrylate monomer that has high strength and low shrinkage. It may explain the color stability of hybrid ceramics by decreasing the polymerization shrinkage and stress so reducing color changes over time. TEGDMA is known for its low viscosity and ability to decrease the curing time but it has been shown to be more prone to leaching out of dental composites compared to other monomers, which can potentially cause discoloration or color shift over time [42–45].

Saba et al. [32] mentioned that increasing the TEGDMA proportion from 0 to 1% will increase the water uptake of Bis- GMA based resins from 3 to 6% respectively. The high wt% of TEGDMA in Vita Enamic will result in increased water sorption and thus allow the penetration of any hydrophilic colorant into the resin matrix [40, 43]. Although, UDMA is hydrophobic compared to BIS-GM, di-methacrylates, can create cross-linked networks that trap unreacted monomers with plasticizers and the formation of a more porous structure. This can increase the water sorption of the material [32]. This may explain the more color changes of Vita Enamic compared to Cerasmart (*p*-value of 0.0120) especially after 12 month of oral environment aging of both materials.

In the same context, Sarikaya et al. [46] found that Vita Enamic had a significantly greater color difference than other resin nano ceramics. Stamenković et al. [47] noticed no differences in the color stability of different materials. Oppositely, Arif et al. [42] mentioned that nanoceramic materials can be best avoided for veneers in areas with high aesthetic requirements especially by coffee drinkers. Also, Al Amri et al. [43] compared color stability of five CAD/CAM materials (Lava Ultimate (LU), Cerasmart (CS), Vita Enamic (VE), Crystal Ultra (CU) IPS e.max (IPS) after soaking in coffee drink resulted in observable color changes except for PICN materials which revealed acceptable color changes but, resin nanoceramic samples has the biggest color differences, with Cerasmat showing greater color changes (ΔE00 = 2.09) than Vita Enamic (ΔE00 < 1.8).

In addition, Materials can experience color changes due to many factors such as abrasion, erosion, exposure to saliva, food, and drinks [44, 48] The ability of a material to withstand occlusal wear is a crucial aspect for the long-term success of a prosthesis. In a study analyzing the wear pattern of resin ceramic matrix, Vita Enamic demonstrated a higher wear pattern compared to Cerasmart. The filler system, including both the type and size, has undergone significant improvements in the mechanical and physical properties of resin matrix materials [16]. Smaller filler sizes resulted in better wear behavior, while larger filler sizes led to a more protruded surface with higher risk of scratching and subsequently more color changes [12, 17]. The manufacturing procedures used to create Vita Enamic and Cerasmart could also have an impact on how stable their colors are. For instance, the structure and properties of the materials may be impacted by the curing conditions, temperature, and pressure used during the manufacturing process [49].

At the end, both groups revealed statistically insignificant variations in color stability, proving that both materials underwent color change. This might imply that both materials’ color stability would be compromised over a longer time frame. Even though the differences may be slight and invisible to patients or clinicians and within clinically acceptable range [10, 11].

### Limitations

The first drawback of this study is the small sample size, which can be rationalized by the fact that it was calculated using the 5% margin of error assumption. Future studies should take lower-margin errors into consideration. Other limitations may be that clinical color change may be impacted by routine toothbrushing with various kinds of toothpaste. It is advised that more research be done to assess the optical characteristics of hybrid materials at various thicknesses and under several aging procedures. A longer-term clinical investigation should confirm these clinical findings.

## Conclusions

1. Cersmart demonstrated better color stability after 1 year of clinical service.
2. Both Vita Enamic and Cerasmart undergo color changes over time within clinical acceptance range.

## Data Availability

The datasets used and/or analysed during the current study are available from the corresponding author on reasonable request. For privacy reasons, however, individual data allowing for the identification of participants cannot be made available.

## Abbreviations

µ: Mean
SD: Standard deviation
CI: Confidence level
Sig.: Significance level
